# Human erythrocytes release ATP by a novel pathway involving VDAC oligomerization independent of pannexin-1

**DOI:** 10.1038/s41598-018-29885-7

**Published:** 2018-07-30

**Authors:** Irene Marginedas-Freixa, Cora Lilia Alvarez, Martina Moras, María Florencia Leal Denis, Claude Hattab, François Halle, Frédéric Bihel, Isabelle Mouro-Chanteloup, Sophie Denise Lefevre, Caroline Le Van Kim, Pablo Julio Schwarzbaum, Mariano Anibal Ostuni

**Affiliations:** 1 UMR-S1134, Integrated Biology of Red Blood Cells, INSERM, Université Paris Diderot, Sorbonne Paris Cité, Université de la Réunion, Université des Antilles, F-75015 Paris, France; 2 Institut National de la Transfusion Sanguine, Laboratoire d’Excellence GR-Ex, F-75015 Paris, France; 3 Instituto de Química y Fisico-Química Biológicas “Prof. Alejandro C. Paladini”, UBA, CONICET, Facultad de Farmacia y Bioquímica, Junín 956, Buenos Aires, Argentina; 4 Universidad de Buenos Aires. Facultad Ciencias Exactas y Naturales, Departamento de Biodiversidad y Biología Experimental, Buenos Aires, Argentina; 5 Universidad de Buenos Aires, Facultad de Farmacia y Bioquímica, Departamento de Química Analítica y Fisicoquímica, Cátedra de Química Analítica, Buenos Aires, Argentina; 6 UMR7200, Laboratoire d’Innovation Thérapeutique, Faculty of Pharmacy, University of Strasbourg, CNRS, 67400 Illkirch Graffenstaden, France; 7 Universidad de Buenos Aires, Facultad de Farmacia y Bioquímica, Departamento de Química Biológica. Cátedra de Química Biológica Superior, Buenos Aires, Argentina

## Abstract

We previously demonstrated that the translocase protein TSPO2 together with the voltage-dependent anion channel (VDAC) and adenine nucleotide transporter (ANT) were involved in a membrane transport complex in human red blood cells (RBCs). Because VDAC was proposed as a channel mediating ATP release in RBCs, we used TSPO ligands together with VDAC and ANT inhibitors to test this hypothesis. ATP release was activated by TSPO ligands, and blocked by inhibitors of VDAC and ANT, while it was insensitive to pannexin-1 blockers. TSPO ligand increased extracellular ATP (ATPe) concentration by 24–59% over the basal values, displaying an acute increase in [ATPe] to a maximal value, which remained constant thereafter. ATPe kinetics were compatible with VDAC mediating a fast but transient ATP efflux. ATP release was strongly inhibited by PKC and PKA inhibitors as well as by depleting intracellular cAMP or extracellular Ca^2+^, suggesting a mechanism involving protein kinases. TSPO ligands favoured VDAC polymerization yielding significantly higher densities of oligomeric bands than in unstimulated cells. Polymerization was partially inhibited by decreasing Ca^2+^ and cAMP contents. The present results show that TSPO ligands induce polymerization of VDAC, coupled to activation of ATP release by a supramolecular complex involving VDAC, TSPO2 and ANT.

## Introduction

Human erythrocytes release ATP following exposure to β-adrenergic stimulation, mechanical deformation, reduced oxygen tension, or acidosis^1^. These stimuli mimic physiological conditions to which erythrocytes are exposed in the vasculature (e.g., when passing through constricted vessels or in contracting striated muscle)^2^. Once released, extracellular ATP (ATPe) can trigger different cellular responses by interacting with purinergic receptors on the cell surface, while its concentration is controlled by the activities of one or more ectonucleotidases^3^. Among the various cellular and systemic responses triggered by RBC-derived ATPe, binding of nucleotides to purinergic receptors on the vascular endothelium results in local generation of vasodilators, which control the vascular calibre^1^.

Depending on the physiological or experimental conditions, RBC-derived ATPe may originate from haemolysis^4^ and/or regulated release by poorly characterized pores, channels or transporters^5–8^. Among these latter molecules is pannexin-1, which is a protein predicted to oligomerize into a transmembrane channel that permeates ATP and small dyes^5,9–11^.

Intracellular signalling routes that trigger regulated ATP release involve activation of heterotrimeric G proteins^10,12–14^. In the Gs pathway, activation of β-adrenergic receptors by various agonists was reported to stimulate adenylyl cyclase, with concomitant increases in the cAMP levels and protein kinase A (PKA) activity^10,15^, whereas direct activation of adenylyl cyclase by forskolin induced parallel increases in cAMP and ATP release in human and rabbit erythrocytes^3^. Additionally, protein kinase C (PKC), which is usually activated by Ca^2+^ and/or diacylglycerol, was shown to modulate the cAMP content and thus affect ATP release from RBCs^16^.

The use of pannexin-1 inhibitors, such as carbenoxolone (CBX) or probenecid (PBC), strongly diminishes the amount of ATP released from RBCs in response to osmotic and shear stress, hypoxia^11^ or adrenergic stimulation^10,17^. Nevertheless, the inhibition exerted by these molecules is never complete, suggesting the existence of additional pannexin-1-independent mechanism(s) of ATP release^10,18^. Indeed, Leal-Denis *et al*.^12^ showed that ATP release from RBCs induced by the peptide Mastoparan-7, a Gi-βγ protein activator, was only partially inhibited by the addition of CBX or PBC. Mathematical modelling of these results was compatible with ATP release by both pannexin-1 and a second conduit, whose nature remained elusive^9,12^.

Studies using RBCs from pannexin-1-deficient mice showed a similar pattern, since ATP release from RBCs exposed to Mastoparan-7^12^ or hypotonic K^+^ solutions^19^ was only partially inhibited in pnx^−/−^ knock out mice compared to pnx^+/+^ mice.

In this context, the voltage dependent anion channel (VDAC) was suggested as an alternative pathway for ATP release^9,15,20^. VDAC is a porin family protein that has been classically described as a mitochondrial protein, although plasmalemmal forms of VDAC have been identified in many cell types (reviewed in^21^), including RBCs^22–24^. Moreover, we have previously demonstrated that three VDAC isoforms are expressed at the RBC membrane in close association with the translocator protein TSPO2 and four isoforms of the adenine nucleotide transporter (ANT)^22,23^.

In fact, TSPO2 and VDAC were shown to be present in similar spectrin-containing cell membrane microdomains but were absent from lipid rafts^23^.

A functional complex of these proteins partially governs anion transmembrane conductance in RBCs^22^. Interestingly, cytosolic ATP is largely anionic in the cytosolic milieu^25^, and various anion channels exhibit a certain degree of permeability to ATP (reviewed in^25^).

In this study, we investigated whether VDAC in close association with TSPO2 and ANT acted as an ATP-release pathway at the RBC membrane and analysed upstream mechanisms involved in VDAC activation. For this purpose, we used different ligands and inhibitors targeting either VDAC, the TSPO-VDAC interface or ANT. We also characterized how protein kinases and oligomerization of VDAC participated in the activation of regulated ATP release from RBCs.

## Results

A previous study proposed that VDAC is one of the conduits promoting ATP release in mature RBCs^15^. We recently identified a macromolecular complex of approximately 800 kDa in the plasma membrane of these cells formed by VDAC, TSPO, ANT and other unidentified proteins, which was activated by TSPO ligands^23^. Then, we aimed to determine whether this complex played an active role in ATP release and investigate the mechanisms involved in this pathway.

### Online ATP release

Online experiments are highly valuable for studies of ATPe kinetics, because the luminometric signal, which is an indirect estimate of the ATPe concentration, can be followed almost continuously at a relatively high acquisition rate. Stimuli are added by diffusion with no media exchange, which minimizes mechanical stress of the RBCs. Since ATPe hydrolysis by ectoATPase activity and ATPe release by haemolysis are negligible under this condition, ATPe kinetics is an almost direct measure of non-lytic ATP release^10^.

**Figure 1:**
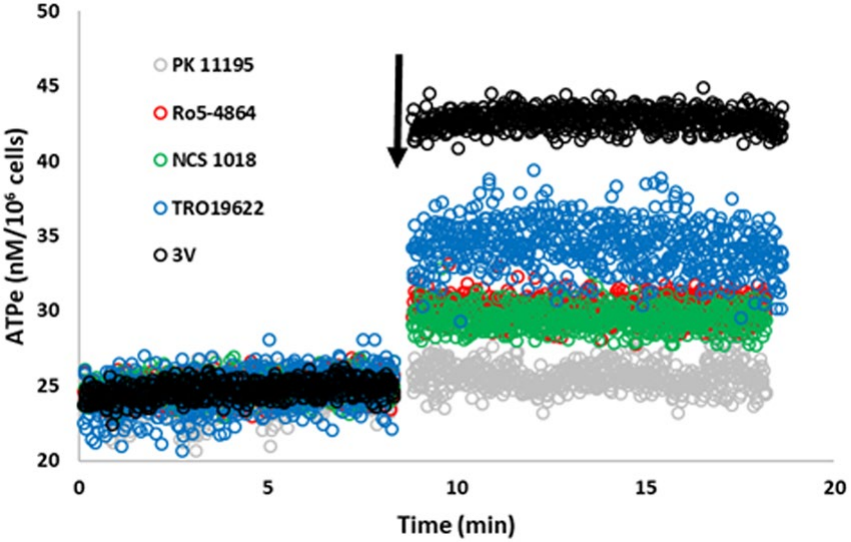
Kinetics of TSPO ligand-induced ATP release. Effect of TSPO ligands on ATP release measured using on line setup. 10 µl of red blood cell suspension (0.75% final haematocrit) were placed on glass coverslip into the luminometer chamber at room temperature. In the time indicated by the arrow, cells were exposed to 30 µM of TSPO ligands PK 11195, Ro5-4864, NCS1018 and TRO19622 or with the adrenergic cocktail 3 V, a cAMP activating cocktail containing 10 mM isoproterenol, 30 mM forskolin and 100 mM papaverine. Mean values of 6–9 independent experiments are shown.

Accordingly, we tested the ability of four different TSPO ligands to induce ATP release from mature RBCs. The ATPe concentrations were continuously recorded throughout the assay. Kinetics curves (Fig. 1) showed a sudden increase in [ATPe] concentration immediately after ligands addition, suggesting fast activation of ATP at a rate of approximately 4.8, 5.5 and 9.2 nM/min/10^6^ cells for NCS1018, Ro5-4864 and TRO19622. [ATPe] reached a plateau at approximately 1-2 min post-stimulus. PK 11195 was not able to induce ATP release. Since –as mentioned above- ATPe hydrolysis by ectonucleotidases is negligible under the experimental conditions^9^, post-activation achievement of a steady level of ATPe reflects inactivation of ATP release.

**Figure 2:**
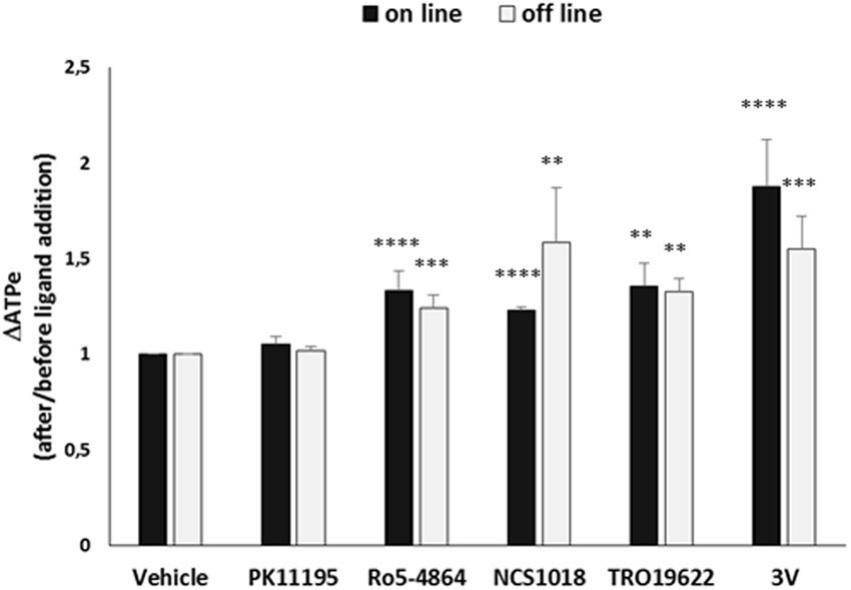
TSPO ligand-induced ATP release. ATP release from human red blood cells was measured using on line (20 °C, 0.75% Haematocrit, black bars) or off line (37 °C, 10% Haematocrit, grey bars) setups as described in material and methods. Values are the ratio between extracellular ATP (ATPe) levels after and before the addition of PK11195 (30 µM), Ro5-4864 (30 µM), NCS1018 (30 µM), TRO196221018 or 3 V (10 µM isoproterenol, 30 µM forskolin, 100 µM papaverin). Results are expressed as means ± SEM and considered different from basal (vehicle, unstimulated) values when **p < 0.01; ***p < 0.001; ****p < 0.0001, n = 6.

The TSPO ligands Ro5-4864, NCS1018 and TRO19622 induced ATP release, increasing ATPe levels of 33, 23 and 35% compared to basal values, respectively (Fig. 2, black bars), whereas PK 11195 had no effect. Conversely, the 3 V cocktail, which is known to cause a strong increase in the cAMP levels^26^, raised [ATPe] by 87%. Control experiments in the presence of the solvents (DMSO or ethanol) used to solubilize the TSPO ligands did not induce significant changes compared to the basal conditions (data not shown) and they were used to calculate ΔATPe (vehicle, black bars Fig. 2).

### Offline ATP release

The online experiments were useful for defining the kinetic features of the ATPe accumulation induced by the TSPO ligands, but these measurements exhibited two main constraints: the cells were held at low haematocrit (0.75%) and at 20 °C, which was the optimal temperature for luciferase activity.

In contrast, the conditions of the offline experiments more closely mimic the *in vivo* situation, where the haematocrit is relatively high and the temperature is 37 °C.

The offline experiments were conducted with the TSPO ligands PK 11195, Ro5-4864, NCS1018 and TRO19622 (Fig. 2, gray bars). These ligands induced the same order of ATP release from the RBCs as the online setup, increasing [ATPe] by 24, 59 and 33%, respectively. The effect was dose-dependent across the concentration range tested (3 to 50 µM, Supplementary Fig. 1). As observed with the online experiments, PK 11195 (30 µM) was not able to induce ATP release. Conversely, exposure to 3 V increased ATPe by 55%. Control experiments in the presence of the solvents (DMSO or ethanol) used to solubilize the TSPO ligands did not induce significant changes compared to basal condition (data not shown) and they were used to calculate ΔATPe (vehicle, gray bars Fig. 2).

### Identification of the conduit for ATP release

**Figure 3:**
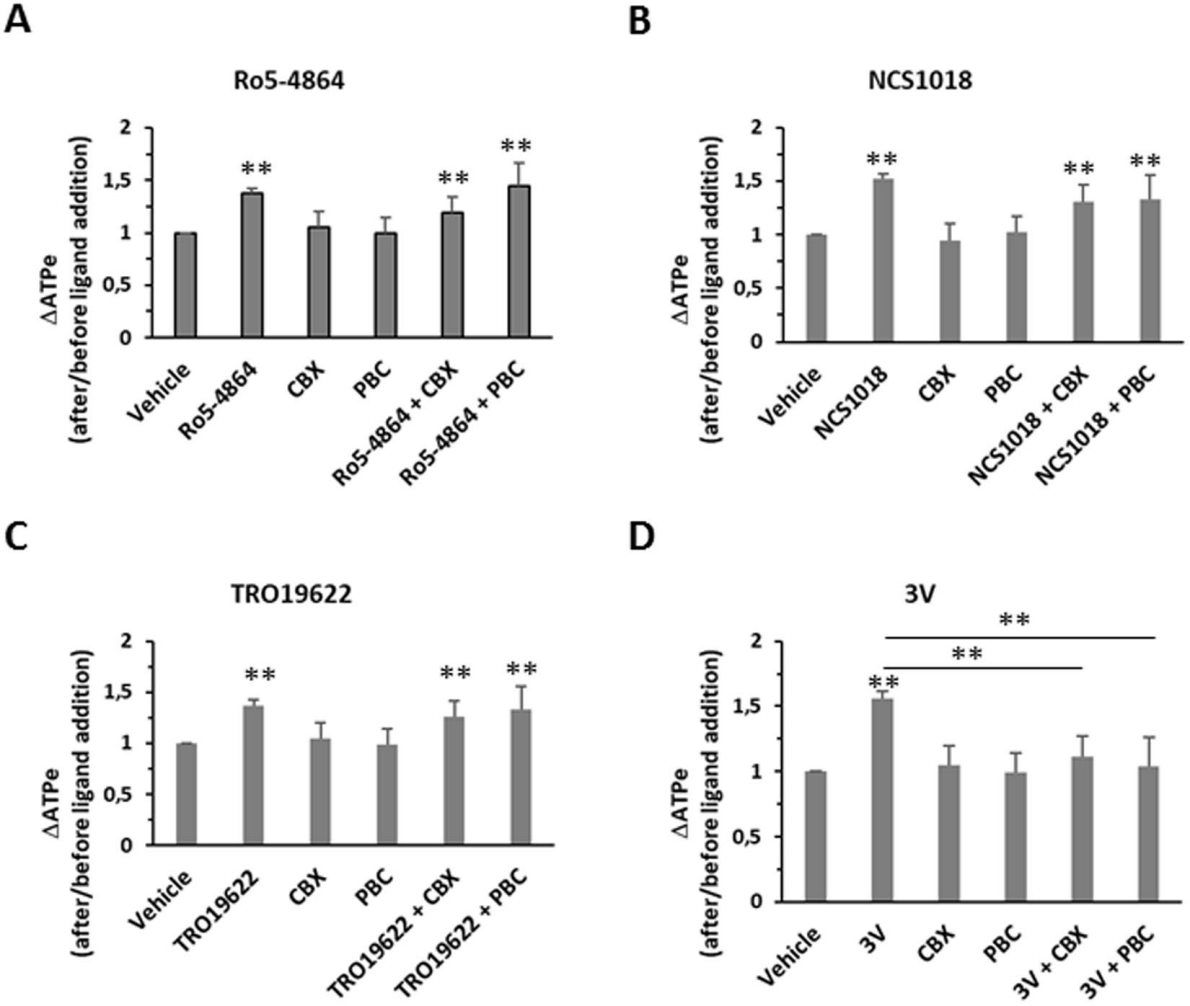
Effect of pannexin-1 inhibitors on TSPO ligands-induced ATP release. RBCs were incubated at 37 °C, 10% final haematocrit. Carbonexolone (CBX, 10 µM) or Probenecid (PBC, 10 µM) were added to human red blood cells (RBCs), alone or before the addition of 30 µM TSPO ligands (Ro5-4864, panel A; NCS1018, panel B; TRO19622, panel C) or adrenergic cocktail 3 V (panel D). Values are the ratio between extracellular ATP (ATPe) levels after and before the addition of different ligands. Results are expressed as means ± SEM and considered different from basal (vehicle, unstimulated) values when **p < 0.01, n = 7. When indicated, differences were significant compared to stimulated condition.

Previous studies identified pannexin-1 as a major conduit for ATP release in RBCs that could be inhibited by CBX and PBC. Under our experimental conditions, 10 µM of both molecules were able to block the ATP release induced by 3 V as previously described^10^(Fig. 2D). However, neither of these compounds repressed the increase in [ATPe] induced by incubation with any of the three TSPO ligands (Fig. 3A–C), suggesting that TSPO-dependent ATP release occurred via a pannexin-1 independent pathway.

**Figure 4:**
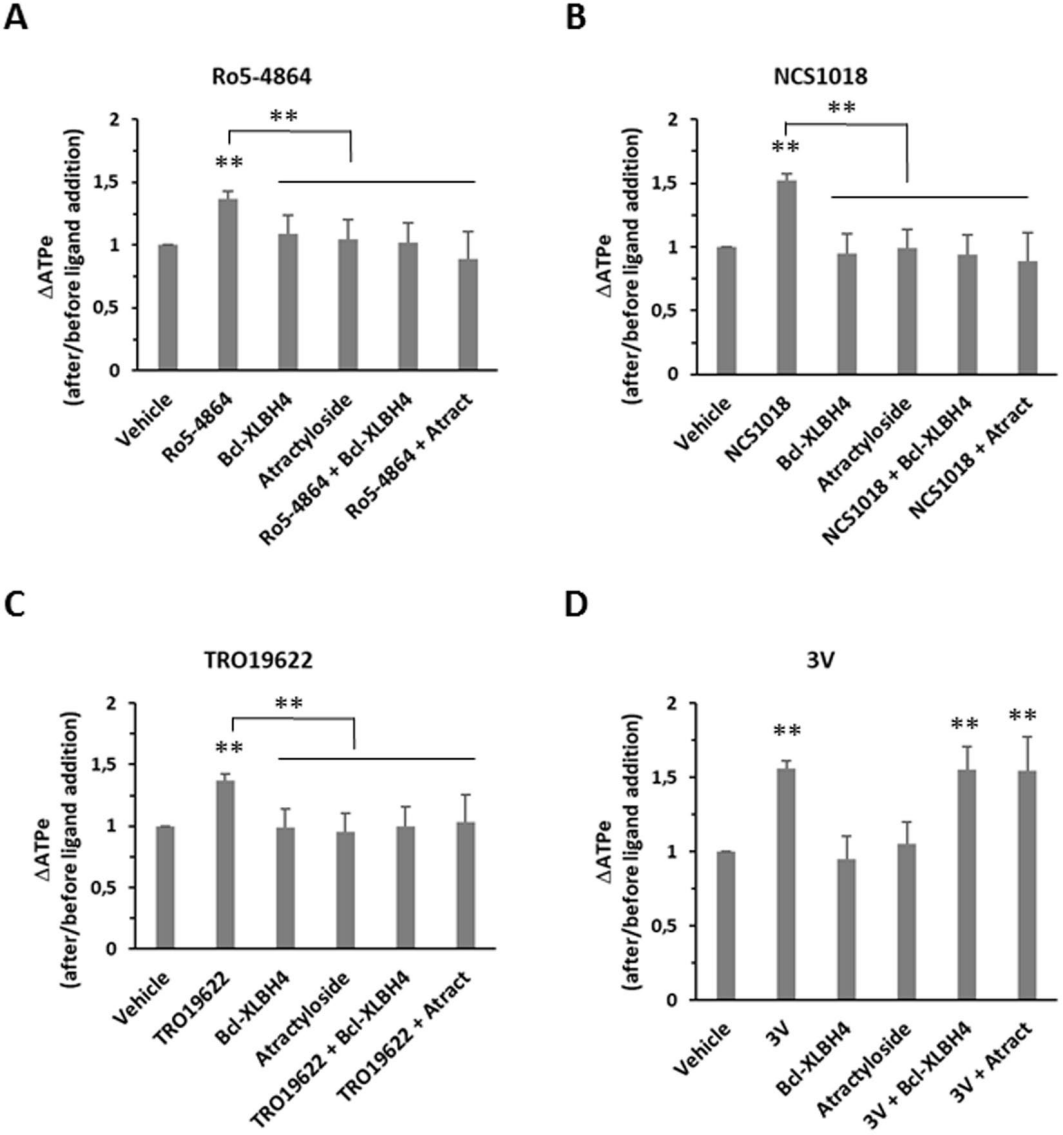
Effect of VDAC and ANT inhibitors on TSPO ligands-induced ATP release. RBCs were incubated at 37 °C, 10% haematocrit. VDAC and ANT inhibitors (Bcl-XLBH4 10 µM and atractyloside 10 µM, respectively) were added to human red blood cells (RBCs), alone or before the addition of 30 µM TSPO ligands (Ro5-4864, panel A; NCS1018, panel B; TRO19622, panel C) or adrenergic cocktail 3 V (panel D). Values are the ratio between extracellular ATP (ATPe) levels after and before the addition of different ligands. Results are expressed as means ± SEM and considered different from basal (vehicle, unstimulated) values when **p < 0.01, n = 6. When indicated, differences were significant compared to stimulated condition.

Because VDAC was previously proposed as an ATP conduit^15^ and taking into account the presence of a multimeric complex with VDAC, TSPO2 and ANT at the surface of RBCs^23^, we used the VDAC inhibitor Bcl-XLBH4 (10 µM) and the ANT inhibitor atractyloside (10 µM) to test whether these partner proteins were involved in the ATP release induced by the TSPO ligands. Following activation by any of the three TSPO ligands, Bcl-XLBH4 and atractyloside (which is also known to interrupt ANT-VDAC interaction^27^), abolished ATP release but did not significantly modify the 3V-induced ATP release (Fig. 4).

### VDAC and ANT polymerization

Previous evidence showed that in mitochondria, VDAC activity was modulated by phosphorylation and polymerization^28–30^, whereas exchanger activity of ANT was strongly regulated by phosphorylation^31^.

**Figure 5:**
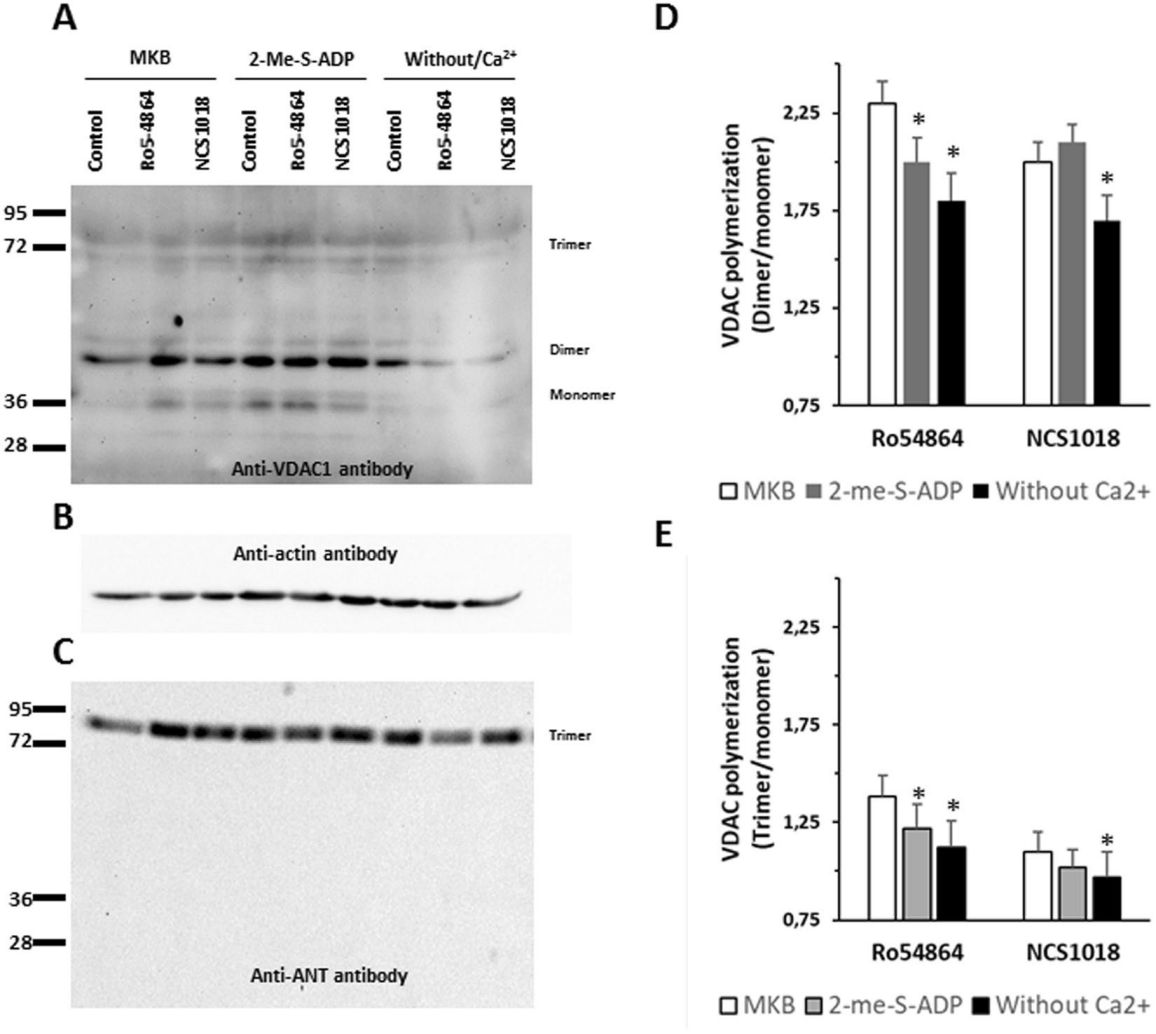
VDAC polymerization is induced by TSPO ligands. Red blood cells (RBCs) were incubated 60 min at 37 °C in 3 different culture media: control (MKB), low cAMP (1 µM 2-Me-S-ADP) and Ca^2+^-depleted (without Ca^2+^). Then they were incubated 10 min at 37 °C in the presence of TSPO ligands Ro5-4864 or NCS1018. Ghost membrane were prepared from each treatment, lysed with 1% Triton X-100 and analysed using an anti-human VDAC1 polyclonal antibody. Monomer, dimer, trimer and tetramer bands of VDAC were identified (Panel A). Anti-b actin antibody was use as loading control (Panel B). Anti-ANT antibody immunolabeling showed only a trimeric band (Panel C). Densities of VDAC immunogenic bands were quantified to calculate dimer/monomer (Panel D) and trimer/monomer (panel E) ratios, under control (MKB, white bars) or experimental conditions (2-Me-S-ADP, grey bars and medium without Ca^2+^, black bars). Results are expressed as means ± SEM of the polymer/monomer ratios, and considered different from basal (control, unstimulated) values when *p < 0.05; n = 4.

Thus, using RBCs membranes we analysed the relative abundance of VDAC oligomers by western blotting (Fig. 5A), using an anti-human VDAC1 antibody. The antibody specificity was confirmed using Jurkat lymphoid cell line, UT7 erythroid cell line and primary CD34+ human progenitors, treated with shRNA targeting VDAC1 (Supplementary Fig. 2). The TSPO ligands enhanced the density of the VDAC dimer and trimer bands (Fig. 5D and E) compared to samples from unstimulated cells. In the presence of Ro5-4864 the densities of the bands increased 2.3 fold (dimers) and 1.38 fold (trimers), while with NCS1018 they increased 2.0 (dimers) and 1.10 (trimers). Since it was previously described that VDAC polymerization was induced by phosphorylation involving the cAMP/PKA and/or Ca^2+^/PKC axes^32,33^, we examined whether changes in cAMP or Ca^2+^ correlated with the appearance of polymeric VDAC immunoreactive bands. Depletion of cAMP (by exposure to 1 µM 2-me-S-ADP) significantly inhibited the appearance of VDAC dimers and trimers induced by Ro5-4864 (Fig. 5D and E). Exposure to Ca^2+^-free medium (i.e., MKB-EGTA medium) led to inhibition of the appearance of VDAC dimers and trimers induced by Ro5-4864 and NCS1018 (Fig. 5D and E). On the other hand, exposure to 3 V in the absence of the TSPO ligands, which increased the cAMP content, led to a 1.48 and 1.6-fold increase in the density of dimers and trimers, respectively (Supplementary Fig. 3).

Western blotting also revealed that ANT is present in a trimeric form. ANT polymerization was neither modified by TSPO ligands, nor by depletion of intracellular cAMP nor extracellular Ca^2+^ (Fig. 5C).

### Roles of PKC and PKA in TSPO ligand-induced ATP release

**Figure 6:**
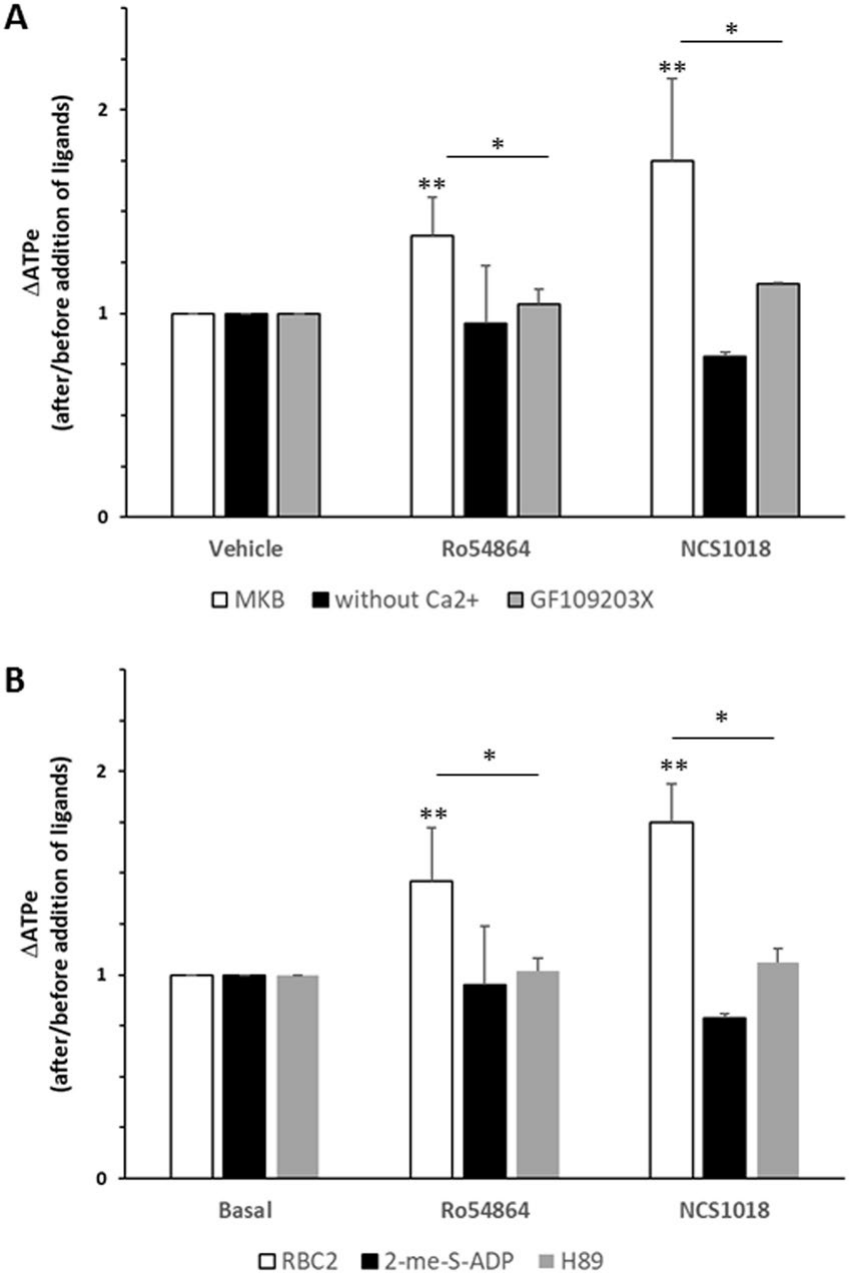
PKC and PKA are involved in ATP release induced by TSPO ligands. Panel A: human red blood cells (RBC) were stimulated by the addition of 30 µM TSPO ligands (Ro5-4864, grey bars or NCS1018, black bars) under control conditions (MKB), in a Ca^2+^-depleted medium (MKB-EGTA) or in the presence of 1 µM of the PKC inhibitor GF109203X. Panel B: RBCs were stimulated by the addition of 30 µM TSPO ligands (Ro5-4864, NCS1018,) under control conditions (MKB, white bars), in a low cAMP medium (1 µM 2-Me-S-ADP, black bars) or in the presence of 10 µM of the PKA inhibitor H89 (H89, grey bars). Results are expressed as means ± SEM and considered different from basal (unstimulated) values when *p < 0.05; **p < 0.01, n = 5. When indicated, differences were significant compared to stimulated condition.

Since phosphorylation in RBCs is governed by PKA and PKC activities, we assessed the action of these enzymes on VDAC-driven ATP release. Addition of 1 µM GF109203X, a well-characterized PKC inhibitor, significantly inhibited the ATP release induced by the TSPO ligands, compared to the control conditions (Fig. 6A). Because PKC activity depends on the Ca^2+^ level, we incubated RBCs in a medium lacking Ca^2+^ and supplemented with the Ca^2+^-chelating molecule EGTA (MKB-EGTA medium). Under these conditions, TSPO ligand-induced ATP release was significantly inhibited (Fig. 6A), suggesting that PKC activation mediated this response.

PKA is activated by the β adrenergic pathway in RBCs, leading to ATP release via an increase in the cAMP content. Thus, the role of PKA activation in the VDAC-dependent ATP efflux was tested under two inhibitory conditions: i) exposure to 10 µM H-89, a specific PKA inhibitor^10^, and ii) exposure to 1 µM 2-me-S-ADP, a potent P2Y13 agonist known to decrease the cAMP content of RBCs^34,35^. Both treatments strongly inhibited the TSPO ligand-induced ATP release (Fig. 6B).

Taken together, these results demonstrated that PKC and PKA are able to modulate TSPO-induced ATP release and suggested that VDAC phosphorylation could be involved in its polymerization.

### Role of Calcium in TSPO ligand-induced ATP release

**Figure 7:**
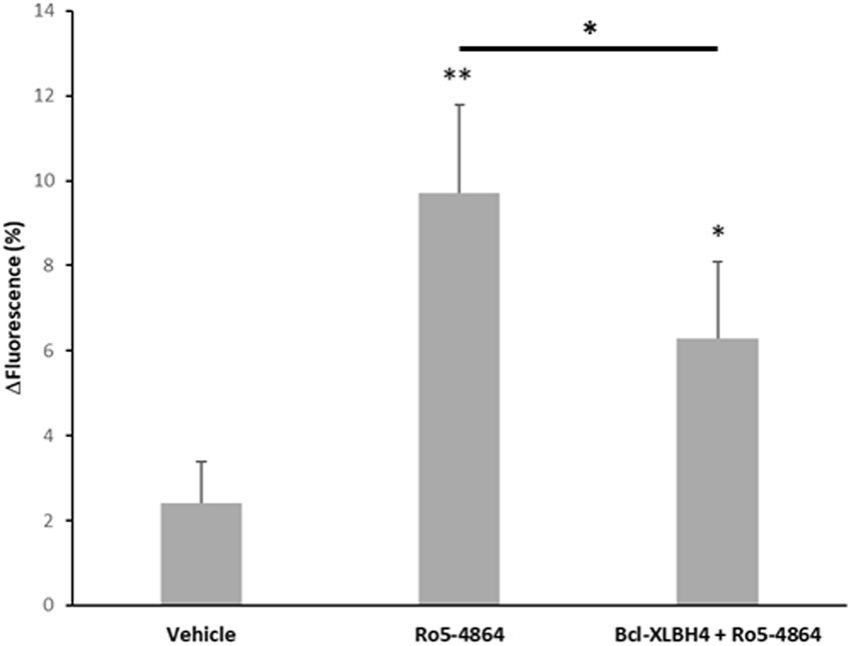
TSPO ligands induced Ca2+ entry in red blood cells. Human red blood cells (RBC) were loaded with the Ca^2+^ probe Fluo4/AM and stimulated with TSPO ligand Ro5-4864 (30 µM) in the presence or in the absence of the VDAC inhibitor Bcl-XLBH4 (10 µM). Control experiments were performed adding equivalent volume of ligand vehicle. Ca^2+^ variations (%) were calculated as the fluorescence ratio (Δfluorescence, Ex: 488 nm; Em: 506 nm) after/before the addition of Ro5-4864. Results are expressed as mean ± SEM and considered different from unstimulated (vehicle) or from stimulated values when *p < 0.05; **p < 0.01, n = 3.

The activities of most PKC isoforms require intracellular Ca^2+^. Because RBCs lack intracellular organelles, increases in intracellular Ca^2+^ are mainly achieved by extracellular Ca^2+^ uptake. Thus, we assessed the ability of the TSPO ligands to induce Ca^2+^ entry. Accordingly, Ro5-4864 slightly but significantly increased intracellular Ca^2+^ 9.4 ± 2.1%. Indeed, addition of the VDAC inhibitor Bcl-XLBH4 (10 µM) inhibited Ro5-464-induced Ca^2+^ entry (Fig. 7).

### Lytic release of ATP

As stated by Dr. Grygorczyk’s group^4,36^, *in vitro* haemolysis of RBCs can significantly contribute to elevation of [ATPe], depending on the experimental conditions. Thus, when analysing ATP release from RBCs, haemolysis has to be assessed to distinguish lytic from non-lytic (regulated) mechanisms of ATP release. As shown in Supplementary Fig. 4, subtracting lytic contribution (derived from haemoglobin content in the supernatant of the samples) from the total ATP (obtained by luminometry), non-lytic ATP could be derived.

As shown in Supplementary Fig. 4, lytic ATP release represented approximately 10–15% of the total ATPe, measured at 37 °C and 10% haematocrit. Importantly, none of the treatments significantly increased the amount of haemolysis from the basal conditions. A previous work suggested – but did not prove – that pannexin-1 inhibitors might decrease haemolysis^4^. Our experiments disproof this possibility under the experimental conditions, because 3V-dependent haemolysis was not altered by pre-treatment with either CBX or PBC (Supplementary Fig. 5). Although a trend towards a decrease in haemolysis was observed under CBX +3 V exposure, these changes were not significant (P > 0.08).

## Discussion

The presence of VDAC-1 in the plasma membrane of several cell types is well established^23,37^. Moreover, in a few cases expression levels or activation of VDAC-1 have been shown to directly correlate with ATP release^15,37^. However, for VDAC and other postulated ATP conduits of the plasma membrane, a recurrent question arises whether these proteins function as single entities or in physical association with other proteins.

In this context, our results demonstrate the existence of a VDAC-TSPO2-ANT complex that mediates ATP release from RBCs. This pathway was specifically activated by TSPO ligands (Ro5-4864, NCS1018 and TRO19622) and blocked by inhibitors of VDAC (Bcl-XLBH4) and ANT (atractyloside), but was insensitive to pannexin-1 blockers (CBX and PBC).

TSPO-dependent changes in [ATPe] were achieved by triggering a strong rate of ATP release, with the efflux increasing from negligible values to approximately 5–10 nM ATPe/min/10^6^ cells during the first min post-stimulus, followed by equally rapid inactivation. This transient nature of the ATP transport process is compatible with the gating features of channels such as VDAC^12^. However, unlike pannexin-1, VDAC, TSPO2 and ANT are not abundant constituents of the RBC membrane^22,23^. In principle, the apparent low transport capacity of the VDAC complex might be compensated by a relatively high kinetic constant of the conduit^22^. Additionally, an approximately 3 order higher intracellular ATP concentration than the observed [ATPe] provides a strong thermodynamic force to drive significant ATP efflux^9,10,12,25^.

PK 11195, TRO19622 and Ro5-4864 are well-characterized ligands of mitochondrial TSPO. However, whereas PK 11195 binds only to TSPO with high affinity, Ro5-4864 and TRO19622 require other mitochondrial proteins, such as VDAC and ANT, for full binding capacity to TSPO^38–40^. Per analogy with TSPO2 in RBCs, we can speculate that these other protein components include VDAC and possibly ANT at the plasma membrane. Thus, Ro5-4864 and TRO19622 will bind to a site localized at the TSPO/VDAC interface^38,39^ and induce the observed activation of ATP release. Conversely, no response was observed with PK 11195, which acts only on TSPO in mitochondria. Noteworthy, PK 11195 was reported to induce TSPO-independent effect when used at high micromolar concentration, both by interaction with other proteins^41^ or by unspecific interaction with the lipid bilayer^42,43^. However, our results showed that micromolar PK 11195 does not affect ATP release from RBCs, further supporting the hypothesis of a specific activity on TSPO-involving complex. Further experiments will be need to address this question as well as to identify the ligands binding sites.

Recently, new pharmacological TSPO ligands belonging to the imidazo[1,2-c]quinazolin-5-one family were reported to improve the production of ATP from neuroblastoma cell lines^44^. From this family, NCS1018 showed a 45-fold lower affinity than Ro5-4864 for heart mitochondrial microsomes, which expressed only the TSPO1 isoform. Thus, a relatively low affinity for TSPO1 but equal potency with Ro5-4864 and TRO19622 for the induction of ATP release agrees well with NCS1018 acting on TSPO2 rather than TSPO1 in RBCs.

Regarding the action of TRO19622, Sridharan *et al*. reported that, in human RBCs, activation of the prostacyclin receptor by iloprost leads to ATP release by VDAC, while TRO19622 specifically inhibited VDAC, thus decreasing ATP exit^15^. This in principle contradicts our results showing an activation effect of TRO19622 on ATP release (Figs 1–4). However, a closer look at the report by these authors shows that, for iloprost induced ATP release, stimulated/basal ratios are around 1.66 and 2.0 for control and TSPO19622-treated cells respectively, indicating that TRO19622 is not inhibiting iloprost action.

On the other hand, TRO19622 was reported to competitively displace specific cholesterol binding to recombinant human TSPO as well as 6-AziP binding to rat neurons VDAC, thus supporting the ability of this compound to bind to TSPO-VDAC interactions sites^38^, as proposed in the present study.

Is noteworthy to mention that TRO19622 is being studied in clinical trials as a potentially useful drug in amyotrophic lateral sclerosis (ALS) treatment. This ligand was administrated to ALS patients treated with riluzol but neither beneficial effect nor adverse event, were demonstrated in patients receiving TRO19622, as compared with those receiving placebo^45^. The doses used in this trial was high (330 mg/patient, daily) and induced ligand accumulation in patient plasma. As in our hands 30 µM TRO19622 induce ATP release from RBCs, ATP plasma levels as well as its impact on purinergic signalling should be carefully studied in future clinical trials.

Activation of ATP release could be brought about by changes in the phosphorylation and/or oligomerization state of plasma membrane VDAC.

Changes in the phosphorylation state of plasma membrane VDAC can be induced by the activities of one or more protein kinases and phosphatases.

The literature concerning plasma membrane VDAC phosphorylation is scarce. Few studies have been exclusively performed on different types of neurons, where exposure to the beta amyloid peptide, hypoxia and hormones^46–48^ appeared to control the phosphorylation state of VDAC, with consequences for cell survival.

Previous reports showed that the cAMP content, which activates PKA, directly correlated with ATP release in RBCs^9,15,49,50^, although other factors affected ATPe regulation independently of cAMP^12,51^.

In this study, PKA-inhibition by H-89, inhibition of cAMP synthesis via activation of P_2_Y_13_ receptors (i.e., exposure to 2-methyl-s-ADP^35^) or pharmacological activation of cAMP synthesis (i.e., exposure to 3 V) correlated directly with changes in the TSPO-induced ATP release. Thus, TSPO ligands probably caused phosphorylation of RBC VDAC by providing a recruitment site for cytosolic PKA, as previously described in another cell model^52^.

Mitochondrial VDAC is present in different oligomeric states, which provide structural and functional anchoring sites for a diverse set of enzymes, transporters and structural proteins that together with VDAC mediate the transport of metabolites between the cytosol and mitochondria (reviewed in^29,53^). The equilibrium between monomers and oligomers is regulated by post-translational modifications, Ca^2+^ levels, reactive oxygen species and/or associated proteins, such as TSPO or Bcl-2-related proteins^29,30^. Interestingly, mitochondrial VDAC was shown to transport ATP as a fully open state oligomer^28^.

In RBCs, TSPO ligands induce the polymerization of VDAC, while inhibition of polymerization correlated with inhibition of ATP release under all treatments and conditions.

Increase of the cAMP content coupled with PKA activation is the main action mechanism of the 3 V cocktail, which induces ATP release mainly via pannexin-1^9^. However, a second CBX-tolerant ATP flux was observed under 3 V treatment^10^. In this context, triggering of VDAC polymerization by 3 V displayed a pattern similar to that induced by the TSPO ligands (Supplementary Fig. 3), strongly suggesting that the VDAC-TSPO interaction accounted for the CBX-insensitive ATP flux activated by 3 V.

Additionally, the slight Ca^2+^ entry induced by the TSPO ligands might have favoured PKC activation, followed by phosphorylation and polymerization of VDAC.

**Figure 8:**
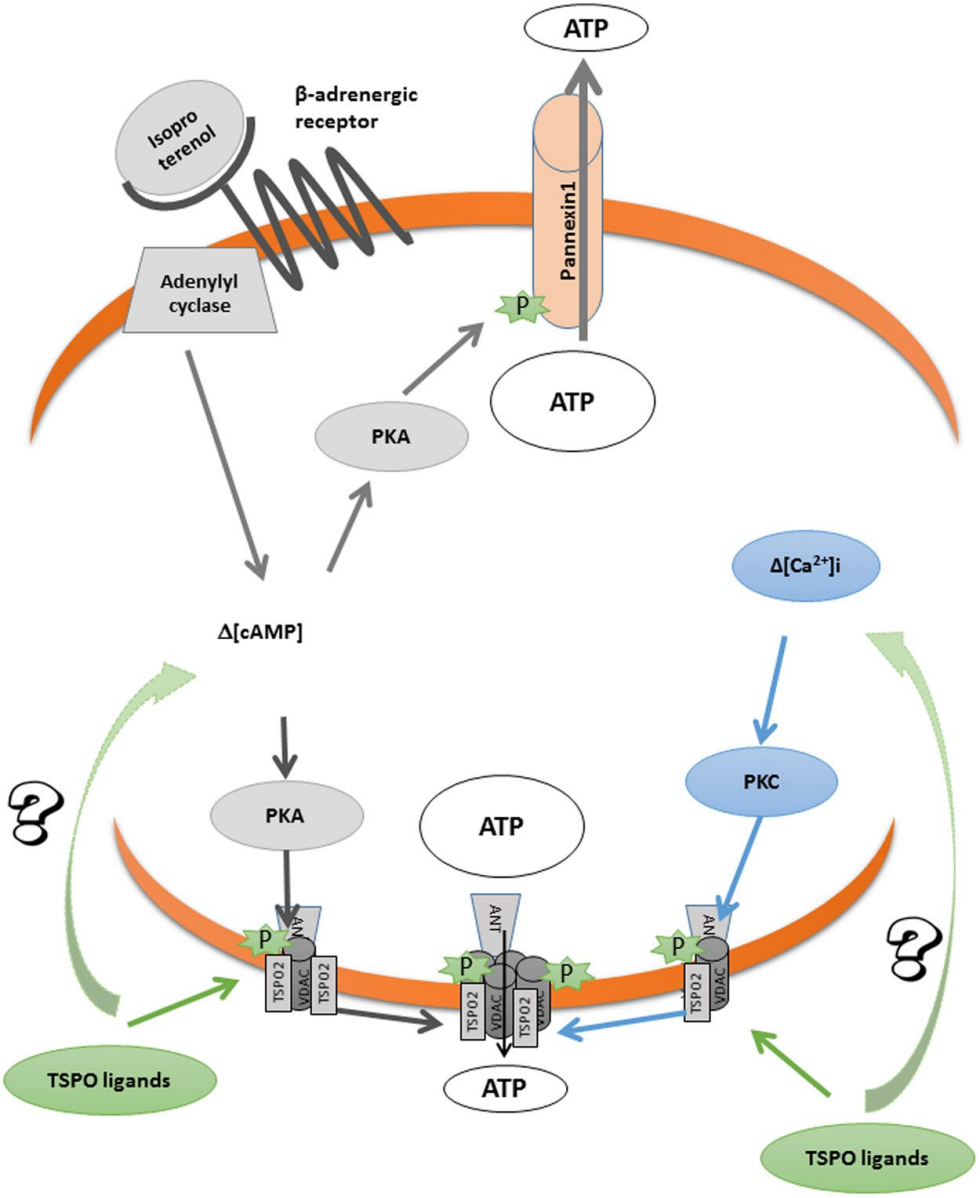
Proposed mechanism for TSPO ligand-induced ATP release. TSPO ligands from benzodiazepines and tricycles families bind to TSPO2/VDAC/ANT complex, inducing calcium entry and, probably enhancing cAMP signalling. Ca^2+^ and cAMP signalling activate PKC and PKA kinases respectively, which induce VDAC phosphorylation and polymerization, allowing ATP release through the polymeric complex. Orange ribbon represents the RBC membrane.

A summary of the results discussed above is illustrated in Fig. 8.

Assuming 30–40% haematocrit in the intravascular space, activation of VDAC in RBCs can be estimated to build up to 1–2 µM [ATPe] in a few minutes. As discussed above, this ATP release is a strictly passive (driven by the electrochemical gradient of ATP) but metabolically active dynamic process. ATP exit is transient and therefore should not impose any energetic burden on RBCs, since it requires only 0.1% of the available cytosolic ATP. This speculation is in line with previous reports suggesting that VDAC should remain closed most of the time^37,54^ and be activated under specific conditions.

*In vivo*, the low micromolar [ATPe] generated by RBC VDAC activity, even under moderate rates of ATPe hydrolysis by different ecto- and exonucleotidases of intravascular cells, would be suitable for activating high affinity P_2_Y receptors of endothelial cells. This step is followed by the production of endothelial-derived vasodilators that relax the smooth muscle cells lining the vessels and ultimately increase the vascular calibre. Studies using RBCs from humans, rabbit and mice support the hypothesis that RBC-derived ATPe is an important determinant of microvascular perfusion^55^ (e.g., under conditions where the vascular tone in the brain, coronary arteries or skeletal muscle is compromised^56^).

In this context, VDAC activation by specific ligands may constitute an important signal modulating RBC-derived ATP release in physiological and pathological conditions. For example, *in vitro P. falciparum* infection of RBCs induced strong activation of ATP release^57^, which *in vivo* should at least partially compensate for the impaired blood flow usually found in malaria patients^58^. The observed increase in [ATPe] in infected RBCs was in part due to the appearance of a CBX-tolerant ATP efflux, which in the context of the present work suggested VDAC activation. Recent findings by our group support this hypothesis by showing that *P. falciparum* induces an increase in RBC membrane conductance through VDAC-like channel activity^23^. Since the VDAC1 expression level has been shown to be upregulated under various pathological conditions^37^, future studies may unravel the extent to which ATP release and VDAC activation are connected.

## Methods

### Red Blood Cell samples

Human blood was obtained by venepuncture from healthy volunteers the day each study was performed. Immediately after blood collection, plasma, platelets and leukocytes were removed by centrifugation (900 × g at 20 °C for 3 min). The supernatant and buffy coat were removed and discarded. The RBCs were resuspended and washed three times in modified Krebs buffer (MKB) (137 mM NaCl, 2.7 mM KCl, 1.5 mM KH_2_PO_4_, 4.72 mM Na_2_HPO_4_, 1.32 mM CaCl_2_, 1.91 mM MgSO_4_, and 5 mM glucose, pH 7.4, 300 mOsM). Packed RBCs were resuspended in MKB supplemented with 0.5% bovine serum albumin (BSA) to the corresponding final haematocrit. Experiments in this study were performed with RBCs purified from fresh blood. This study was approved and conducted according to institutional ethical guidelines of the National Institute for Blood Transfusion (INTS, Paris, France). All procedures were carried out in accordance to the Declaration of Helsinki. Written informed consent was given by the donors.

### Chemicals

Ro5-4864, PK 11195, 2-methylthioadenosine 5-diphosphate (2MeS-ADP, P2Y13 agonist), H89 (PKA inhibitor), BCL-xL BH4 (VDAC inhibitor), atractyloside (ANT inhibitor), carbenoxolone (CBX), probenecid (PBC), firefly luciferase (EC 1.13.12.7), forskolin, isoproterenol, papaverine, and ethylene glycol tetraacetic acid (EGTA) were purchased from Sigma-Aldrich (Marne la Coquette, France). GF109203X (PKC inhibitor) was purchased from Tocris Bioscience (Bristol, UK). D-Luciferin and Fluo-4/AM were purchased from Life Technologies (Saint Aubin, France). TRO19622 (Cholest-4-en-3-one oxime) was purchased from TROPHOS (Marseille, France)^38^. N,N-Diethyl-2-(2-(furan-2-yl)-5-oxomidazo[1,2-c]quinazolin-6(5H)-yl)acetamide (NCS1018), which belongs to the imidazo[1,2-c]quinazolin-5-one family, was synthesized following the previously described protocol^44^.

### Online and offline ATPe measurements

ATPe was measured by quantitative luminometry using firefly luciferase, which catalyses the oxidation of luciferin in the presence of ATP to produce light^59,60^. The ATPe concentration was determined continuously (online) or at discrete times (offline). Changes in [ATPe] over time were used to estimate ATP release from RBCs.

In online experiments, 10 µl of RBCs at 3% haematocrit were incubated at 20 °C with 30 µl of a luminometry mix (MKB medium containing 0.01 µM luciferase, 0.2 mM luciferin, and 0.1 mg/ml of Coenzyme A). The cells were mounted in the assay chamber of a custom-built luminometer as previously described^59^. Light emission was quantified continuously under the different treatments. At the end of the incubations, the time course of light emission was transformed into the ATPe concentration versus time using a calibration curve, with ATP stock solutions ranging from 1 µM to 50 µM. This method allows real-time kinetic measurements but is limited by low haematocrit and low temperature conditions.

Once optimal time windows were determined, we performed offline experiments under experimental conditions close to physiological ones. I.e., the haematocrit was adjusted to 10% and the samples were incubated 10 min at 37 °C in the presence of the ligands. At discrete times, samples were centrifuged at 450 × g for 3 min at RT. The supernatants were stored at −20 °C prior to the analysis. Then, 4.5 µl of the supernatant samples were added to 45 µl of the luminometry mix and placed in the assay chamber of the luminometer to determine the light intensity. Calibration of the luminometric signal was similar to that of the online experiments. The results are expressed as [ATPe] (nM) or ΔATPe (i.e., the ratio of [ATPe] before and after the addition of a given stimulus).

The increase in [ATPe] due to regulated ATP release was calculated by taking the experimentally measured [ATPe] and subtracting the estimated [ATPe] due to haemolysis. Except where otherwise stated, results of ATP release experiments reflect the ATP release from non-lytic origin.

### Measurement of haemolysis and estimation of lytic ATPe

Haemolysis leads to haemoglobin (Hb) release into the extracellular milieu, where the Hb content can be quantified by monitoring light absorption at 405 nm. Using this procedure, we previously observed that the amount of Hb was proportional to the amount of lysed RBCs^9^.

In the offline measurements, paired samples were taken to assess ATPe and haemolysis. The Hb content of the samples was determined as described above. Then, the amount of lytic ATP contributing to [ATPe] was estimated from the total amount of haemolysed cells (calculated as described above) and the intracellular ATP content of the RBCs, which under the experimental conditions was 148 pmol ATP per 10^6^ RBCs as previously reported by our group^12^.

### Incubation media

In most experiments, the RBCs were suspended in MKB medium. However, in the measurements using Ca^2+^-depleted medium, the cells were suspended in MKB without CaCl_2_ and supplemented with 0.1 mM EGTA and 2.5 mM MgSO_4_ (MKB-EGTA).

### ATP release inducers

The TSPO ligands PK 11195, Ro5-4864, TRO19622 and NCS1018 were used at 3, 10, 30 and 50 µM concentrations. Their chemical formulas are shown in Supplementary Fig. 6. These ligands are well characterized as highly specific for TSPO1 isoform (nM range of affinity). However, Fan *et al*. reported that the mammal TSPO2 isoform has very less affinity for PK 11195 than TSPO1^61^. Indeed, we have previously demonstrated that human RBC was able to bind Ro5-4864 showing an affinity value of 1.5 ± 0.6 µM^23^.

The new synthetic ligand NCS1018, was able to displace well known radiolabelled ligands [^3^H]PK 11195 and [^3^H]Ro5-4864 from rat heart microsomes enriched in TSPO1with IC_50_ values of 260 and 580 nM, respectively^44^.

3 V, which is an adrenergic cocktail containing 10 µM isoproterenol (beta adrenergic agonist), 30 µM forskolin (adenylyl cyclase activator) and 100 µM papaverin (inhibitor of phosphodiesterases), was used to induce an acute and huge increase in intracellular cAMP leading to ATP release in RBCs as previously described^26^.

### Inhibitors

The cells were exposed to inhibitors before the addition of the ATP inducers as follows:

1 µM GF109203X (PKC inhibitor), 10 µM H-89 (PKA inhibitor), 10 µM atractilosyde (ANT inhibitor), or 10 µM BCL-XLBH4 (VDAC inhibitor).

As previously described, 1 µM 2-Methyl-s-ADP was used as an inhibitor of cAMP synthesis, since binding of this ligand to P2Y13 leads to activation of Gi coupled to inhibition of adenylate cyclase activity^35^.

The addition of 10 µM carbenoxolone (CBX) or 10 µM probenecid (PBC) was used to inhibit pannexin-1 as previously reported^10^. Since CBX and PBC are not strictly specific for pannexin-1^62^, several issues have to be taken into consideration to ensure specific blockage of this protein: (i) similar effects of CBX and PBC (two chemically dissimilar compounds) on ATP release should be obtained and (ii) a relatively low CBX concentration should be used (e.g., exposure to ≤10 µM CBX is known to block pannexin-1 but not connexins)^63^.

### TSPO ligand-induced polymerization assays

One ml of washed cells was incubated 10 min at 37 °C in the presence of 30 µM of TSPO ligands (the same conditions used to measure ATP release). Immediately after incubation, the cells were centrifuged at 450 x g for 3 min at RT and suspended in an isosmotic buffer 5P8 (5 mM NaH_2_PO_4_; 0.35 mM EDTA, pH 8, containing 1 mM phenyl methane sulfonyl fluoride, PMSF) to prepare ghost membranes as previously described^23^. Then, fresh ghost membranes were suspended into a lytic buffer containing 150 mM NaCl, 1% glycerol, 5 mM EDTA, 20 mM Tris HCl (pH 7.4), 1% Triton X-100, and a protease inhibitor cocktail. Ghosts’ lysis was performed at 4 °C, and the samples were stored at −20 °C prior to SDS-PAGE and the Western Blot analysis.

### SDS-PAGE and Western Blotting

Samples of Triton X-100-lysed ghost membranes (30 µg) were run (90 min at 180 V, room temperature) in a SDS-polyacrylamide gel (10%, 1 mm thickness) under reducing conditions (570 mM βME). Running buffer: 250 mM Tris, 192 mM glycine, 5% SDS, pH = 8.3. Transfer to nitrocellulose membranes (90 min at 30 V, room temperature) was performed in transfer buffer containing 12.5 mM Tris, 96 mM glycine, 20% ethanol, 0.1% SDS, pH = 8.5. Immunoblotting was performed using rabbit anti-hVDAC1 antibody (Santa Cruz Biotechnology, B-6, dilution 1:1000), or rabbit anti-ANT polyclonal antibody (a generous gift from Dr G. Brandolin, CNRS, CEA, Grenoble, France, dilution 1:1000^64^). Western blotting secondary labelling was performed with an anti-rabbit IgG secondary antibody (Jackson ImmunoResearch, dilution 1:5000). Loading control was performed using HRP Conjugated anti-β-Actin mAb (Cell Signaling Technology, 13E5, dilution 1:3000).

### Ca^2+^ measurements

Washed RBCs at 1% haematocrit were incubated in the presence of 5 µM Fluo-4/AM for 1 h at 37 °C. Then, the samples were washed and diluted to 0.25% haematocrit in the same buffer. Ca^2+^ entry was induced by the addition of the TSPO ligand Ro5-4864 in the presence of the VDAC inhibitor Bcl-XLBH4 (10 µM). Ca^2+^ variation was quantified by fluorescence (Ex: 488 nm; Em: 506 nm).

### Statistical Analysis

In each graph, the data represent the mean ± standard error of the mean (SEM) unless otherwise noted. If indicated, statistical significance was calculated using one-way analysis of variance followed by Dunnett’s multiple comparison test with the GraphPad Prism software (Graphpad Software, CA, USA). Differences were considered significant when p < 0.05.

## Electronic supplementary material

Supplementary Figures
