# Supplementary material for: Human erythrocytes release ATP by a novel pathway involving VDAC oligomerization independent of pannexin-1
Source: Sci Rep. 2018 Jul 30;8:11384. doi: 10.1038/s41598-018-29885-7 (PMC6065367; doi:10.1038/s41598-018-29885-7)
Supplement: Supplementary file 1 — Supplementary Figures [file 41598_2018_29885_MOESM1_ESM.pdf]

Human erythrocytes release ATP by a novel pathway involving VDAC oligomerization independent of pannexin-1

Irene Marginedas-Freixa[1,2], Cora Lilia Alvarez[3,4], Martina Moras[1,2], María Florencia Leal Denis[3,5], Claude Hattab[1,2], François Halle[6], Frédéric Bihel[6], Isabelle Mouro-Chanteloup[1,2], Sophie Denise Lefevre[1,2], Caroline Le Van Kim[1,2], Pablo Julio Schwarzbaum[3,7] and Mariano Anibal Ostuni[1,2]

Authors' affiliation :

[1]UMR-S1134, Integrated Biology of Red Blood Cells, INSERM, Université Paris Diderot, Sorbonne Paris Cité, Université de la Réunion, Université des Antilles, F-75015 Paris, France.
[2]Institut National de la Transfusion Sanguine, Laboratoire d'Excellence GR-Ex, F-75015 Paris, France.
[3] Instituto de Química y Fisico-Química Biológicas "Prof. Alejandro C. Paladini", UBA, CONICET, Facultad de Farmacia y Bioquímica. Junín 956. Buenos Aires, Argentina.
[4] Universidad de Buenos Aires. Facultad Ciencias Exactas y Naturales. Departamento de Biodiversidad y Biología Experimental. Buenos Aires, Argentina.
[5] Universidad de Buenos Aires. Facultad de Farmacia y Bioquímica. Departamento de Química Analítica y Fisicoquímica. Cátedra de Química Analítica. Buenos Aires, Argentina.
[6]UMR7200, Laboratoire d'Innovation Thérapeutique, Faculty of Pharmacy, University of Strasbourg, CNRS, 67400 Illkirch Graffenstaden, France.
[7] Universidad de Buenos Aires. Facultad de Farmacia y Bioquímica. Departamento de Química Biológica. Cátedra de Química Biológica Superior. Buenos Aires, Argentina.

Corresponding Author:
Mariano Anibal Ostuni
UMR-S1134, Integrated Biology of Red Blood Cells
6, rue Alexandre Cabanel. 75015 Paris, France.
Phone: +33 1 4449 3135
Fax: +33 1 43065019

Sup Fig 1 Dose-response of TSPO ligands on ATP release

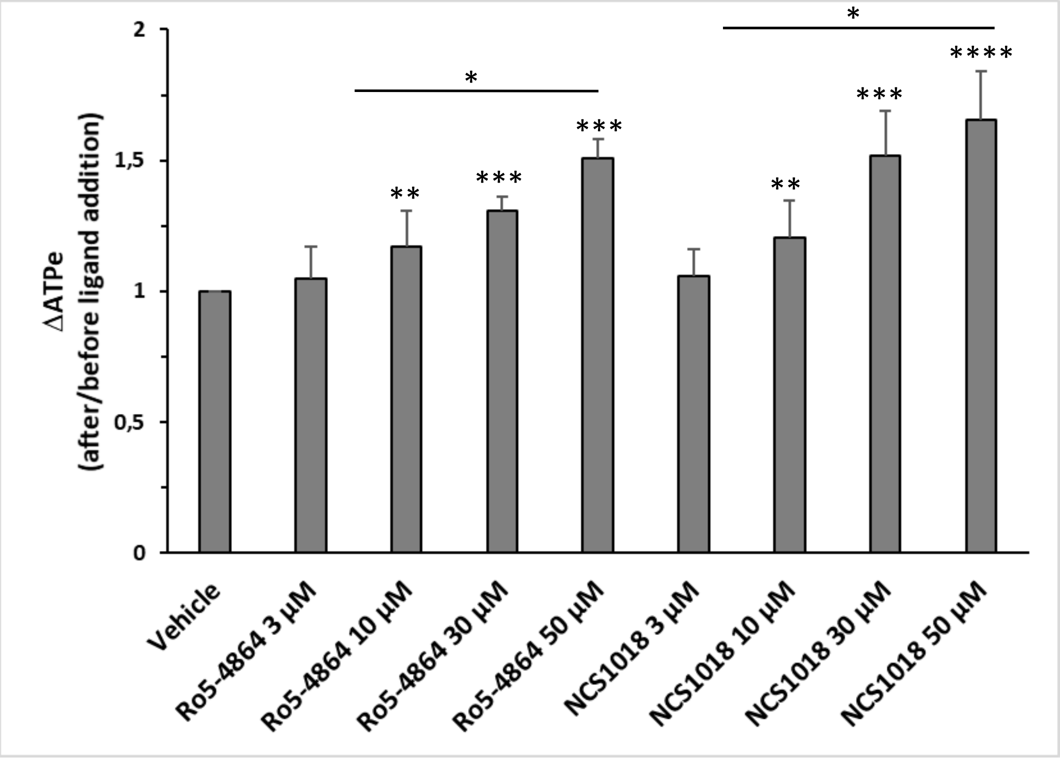

Supplementary Figure 1. Dose-response of TSPO ligands on ATP release measured using off line setup. Red blood cell suspensions (10% final haematocrit) were stimulated (10 min at 37 °C) with TSPO ligands Ro5-4864 or NCS1018. 6 independent experiments were performed by triplicate. Results are expressed as mean ± SEM and considered different from vehicle (addition of equal volume of DMSO or Ethanol) values when *p<0,05; **p<0.01; ***p<0.001; ****p<0.0001, n=6.

Fig Sup 2. Specificity of anti-hVDAC1 polyclonal antibody

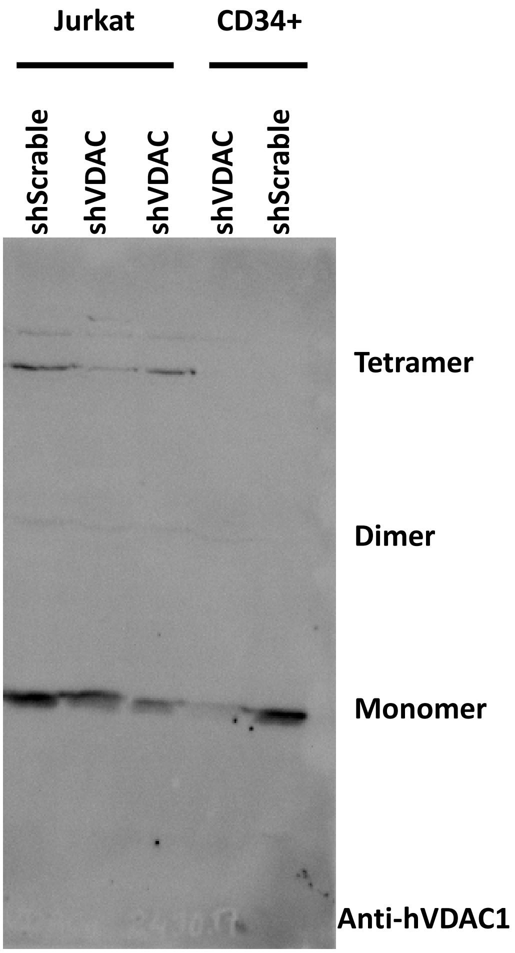

Supplementary Figure 2. Specificity of anti-hVDAC1 polyclonal antibody. Antibody' sspecificity was tested using human lymphoid (Jurkat), erythroid (UT7) and primary CD34+ cells, transfected with scramble shRNA or anti VDAC1 shRNA. Monomer, dimer and tetramer bands of VDAC1 were identified in Jurkat cells. CD34+ and UT7 cells showed only monomer. Immunoblotting shows that in the 3 cells types, shVDAC significantly reduced both monomer and polymer expression.

Fig Sup 3. VDAC polymerization by 3V

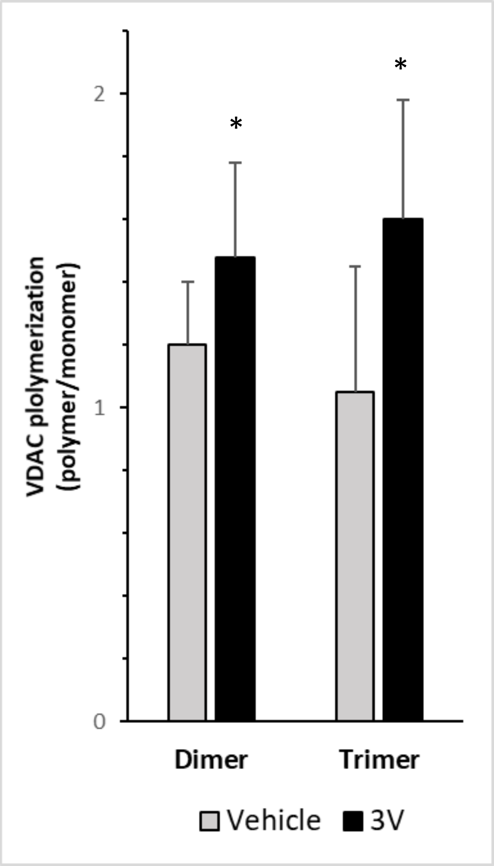

Supplementary figure 3. VDAC polymerization is induced by adrenergic cocktail 3V. Red blood cells (RBCs) samples were incubated at 37 °C and stimulated with adrenergic cocktail 3V. Then ghost membranes were obtained by hypo-osmotic treatment and lysed with 1% Triton X-100. Proteins were separated by SDS-PAGE under non-reducing conditions and labelled using an anti-human VDAC polyclonal antibody. Bands densities were quantified to calculate dimer/monomer and trimer/monomer ratios. Results are expressed as mean ± SEM; n=3; *p<0,05

Fig Sup 4. Lytic vs Non lytic ATP release

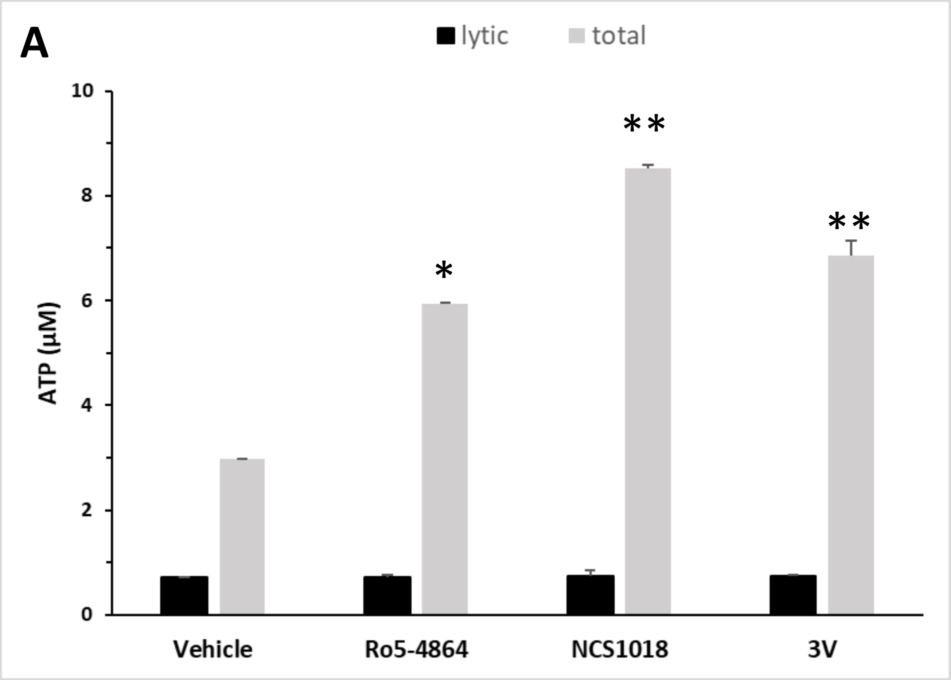

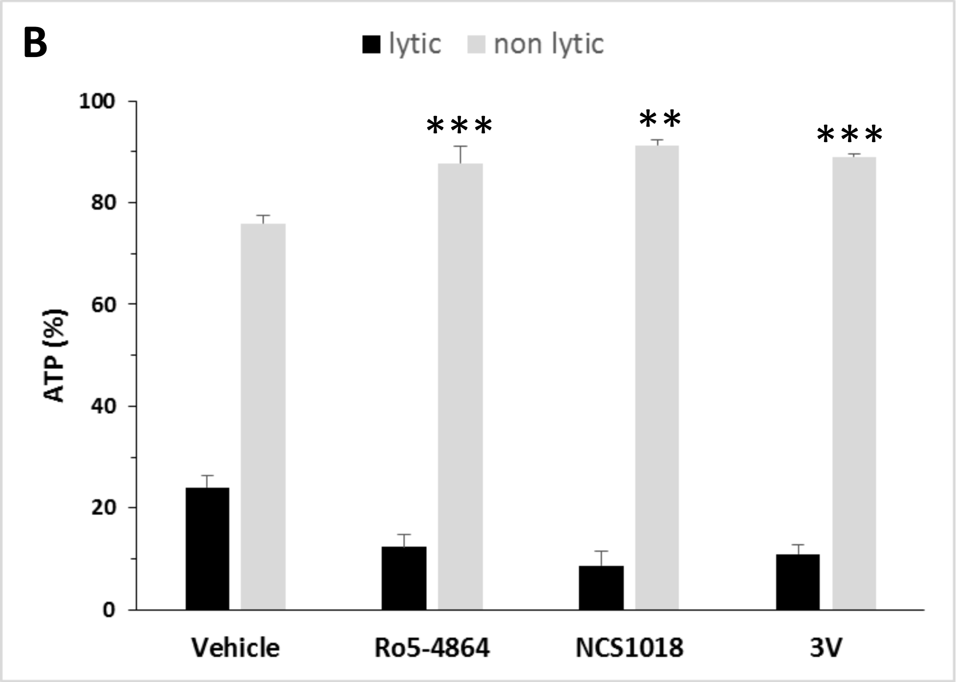

Supplementary Figure 4. TSPO ligands induced non-lytic ATP release. Human red blood cells (10% final haematocrit) were incubated at 37 °C and stimulated with different TSPO ligands or the adrenergic cocktail 3V. After each experiment, total ATP content and haemolysis were measured as described in material and methods section. A) Total (grey bars) and lytic (black bars) ATP after ligands addition are shown. B) Lytic (black bars) and non-lytic (grey bars) ATP after ligands addition, expressed as percentage of total ATP (lytic + non lytic). Results are expressed as mean ± SEM, n=5, and were considered different from vehicle when *$p<0,05$; **$p<0.01$; ***$p<0.001$

Fig Sup 5. Pannexin-1 inhibition and haemolysis

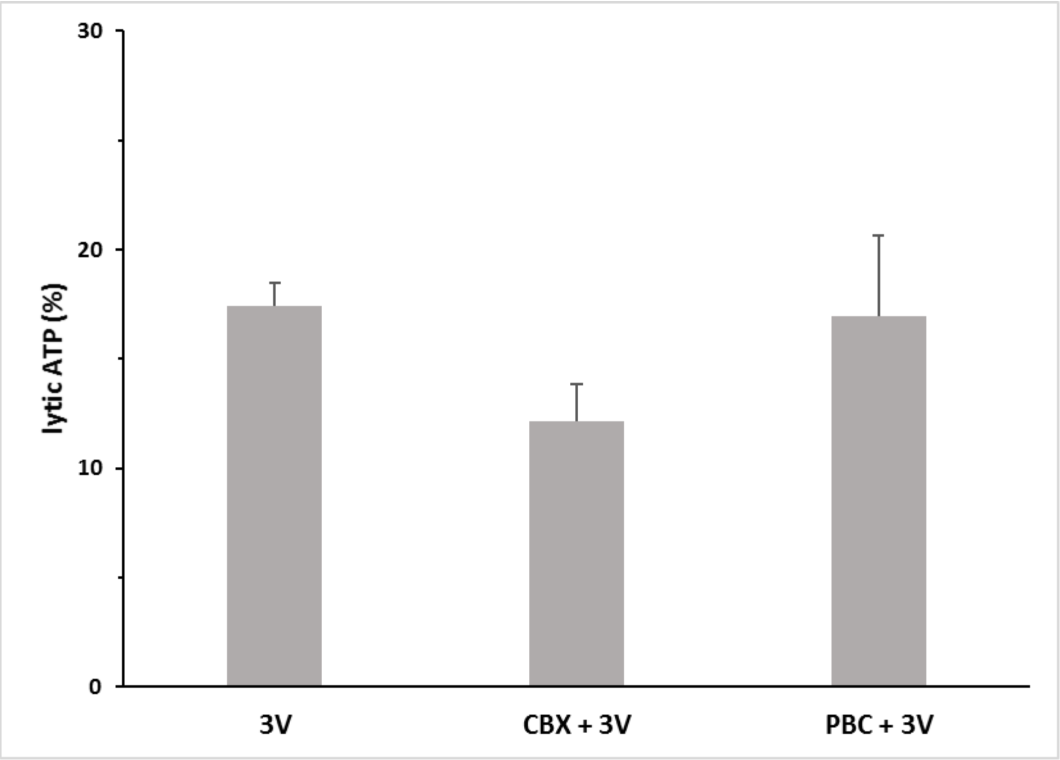

Supplementary Figure 5. Pannexin-1 inhibitors do not modify 3V-induced haemolysis. Human red blood cells (10 % final haematocrit) were incubated at 37 °C and pre-stimulated with different pannexin-1 inhibitors carbonexolone (CBX, 10 µM) or probenecid (PBC, 10 µM) 10 min before the addition of the adrenergic cocktail 3V. Values represent the % of ATP from haemolytic origin after de addition of 3V. Results are expressed as mean ± SEM, n=3

Sup Fig 6 Chemical structure of TSPO ligands

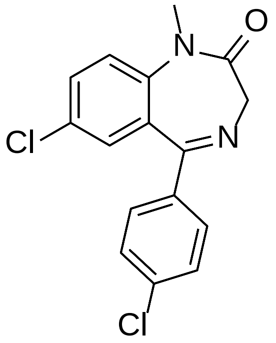

Ro5-4864

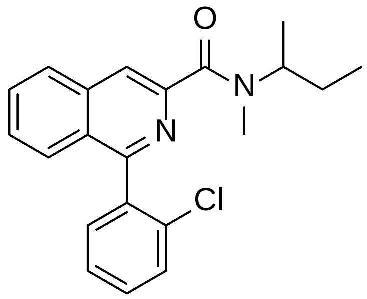

PK11195

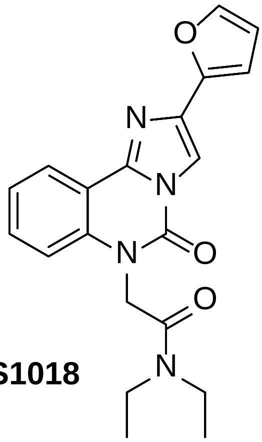

NCS1018

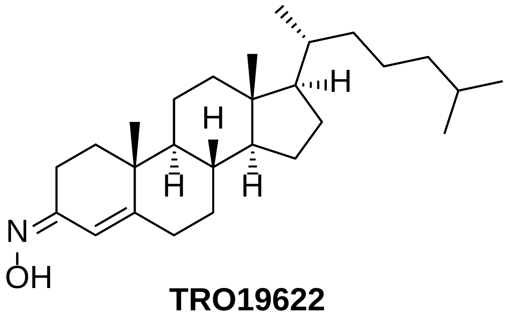

TRO19622

Supplementary Figure 6. Chemical structure of TSPO ligands. Structure of the four TSPO ligands used in this study.
